# Symptomatic Vulvar Primary Cutaneous Amyloidosis Associated With Low–Risk HPV: A Case Report

**DOI:** 10.1002/ccr3.71966

**Published:** 2026-01-28

**Authors:** Jorge Hoegl, Andreina Fernandes, Daniel Marquez, Ysleyer Silva

**Affiliations:** ^1^ Cátedra de Ginecología, Escuela de Medicina José María Vargas, Facultad de Medicina Universidad Central de Venezuela Caracas Venezuela; ^2^ Centro Clínico Profesional Caracas Unidad de Ginecología Oncológica Caracas Venezuela; ^3^ Laboratorio de Genética Molecular Instituto de Oncología y Hematología, MPPS Caracas Venezuela; ^4^ Instituto de Investigaciones Odontológicas Raúl Vincentelli, Facultad de Odontología Universidad Central de Venezuela Caracas Venezuela; ^5^ Hospital Universitario de Caracas Unidad de Perinatología Dr. Freddy Guevara Zuloaga Caracas Venezuela; ^6^ Unidad de Atención Femenina (UNIFEM) Caracas Venezuela; ^7^ Laboratorio R&S Diagnósticos Caracas Venezuela

**Keywords:** case report, cutaneous, HPV, nodular amyloidosis, vulva

## Abstract

Primary localized cutaneous amyloidosis (PCLA) of the vulva is an infrequent diagnosis. Its clinical presentation may mimic neoplastic or inflammatory lesions and could even be associated with human papillomavirus (HPV) of low or high oncogenic risk, making accurate diagnosis and exclusion of systemic involvement essential. A 73‐year‐old woman presented with a flat, macular, solitary vulvar lesion. Histologic evaluation revealed amyloid deposits in the papillary dermis, confirmed by Congo red staining and apple‐green birefringence. HPV genotyping identified type 40, a low‐risk genotype, with no evidence of intraepithelial neoplasia. The diagnosis of systemic amyloidosis was ruled out by a multidisciplinary team evaluation. A complete local excision was performed. After 33 months of follow‐up, the patient remains in remission. This case highlights the importance of including PCLA in the differential diagnosis of vulvar lesions and suggests a potential role of HPV as a possible trigger for vulvar localized amyloidogenesis.

## Key Clinical Message

Primary vulvar cutaneous amyloidosis is a rare non‐neoplastic lesion that should be considered in differential diagnoses, with HPV infection potentially contributing to amyloid deposition even without VIN. Surgical resection may be an alternative in select cases, though systemic amyloidosis must first be excluded.

## 1 Introduction

Amyloidosis encompasses a group of disorders caused by abnormal protein metabolism, resulting in the extracellular deposition of amyloid, a fibrillar proteinaceous substance. It is classified based on systemic involvement, the presence of underlying diseases, patient characteristics, and the biochemical nature of the deposits [1]. The primary categories include systemic or localized forms, primary or secondary types, and hereditary variations [2].

Primary localized cutaneous amyloidosis (PLCA) is rare, with its incidence varying among populations. In Asia, the estimated prevalence is approximately 0.98 per 10,000 individuals, which is higher than the reported frequencies in Western populations, where data remain limited [3, 4]. PLCA is characterized by amyloid deposition confined to the skin, typically the papillary dermis, without systemic involvement [5]. These deposits display classical features such as congophilia and apple‐green birefringence under polarized light [6]. PLCA can occur independently or in association with benign or malignant lesions. It includes three subtypes: macular, lichenoid, and nodular, the latter being the rarest [5].

Primary vulvar cutaneous amyloidosis is an extremely uncommon presentation, and most of the reported cases associated with HPV infection involve usual‐type vulvar intraepithelial neoplasia (VIN) and high‐risk genotypes, particularly HPV 16 or 51. To date, reports of vulvar PLCA occurring in the absence of VIN and linked to low‐risk HPV genotypes are minimal [5, 7]. This case adds novel evidence suggesting that low‐risk HPV infection without epithelial dysplasia may represent a non‐neoplastic pathway of amyloidogenesis, broadening the clinical spectrum of HPV‐associated vulvar lesions and reinforcing the importance of considering PLCA in patients with atypical macular vulvar lesions. Clinically, it may mimic inflammatory or neoplastic vulvar lesions [1]. Here, we present a rare case of vulvar PLCA associated with human papillomavirus (HPV). Despite its low incidence, accurate differentiation from other dermatologic and neoplastic conditions of the vulva is crucial, as its management and prognosis differ significantly.

## 2 Case History

A 73‐year‐old woman presented for gynecologic evaluation in June 2022. She presented a vulvar lesion of at least 2 months of evolution, associated with pruritus and intermittent mild stabbing pain, with no other additional findings. She had a history of pulmonary tuberculosis and basal cell carcinoma in the left nasolabial region. She was a G3, P3, asymptomatic postmenopausal woman with no other significant medical history or systemic diseases. No sexual intercourse for more than a decade. She was afebrile, hydrated, hemodynamically stable, and had no other systemic symptoms or dermatological lesions.

**FIGURE 1:**
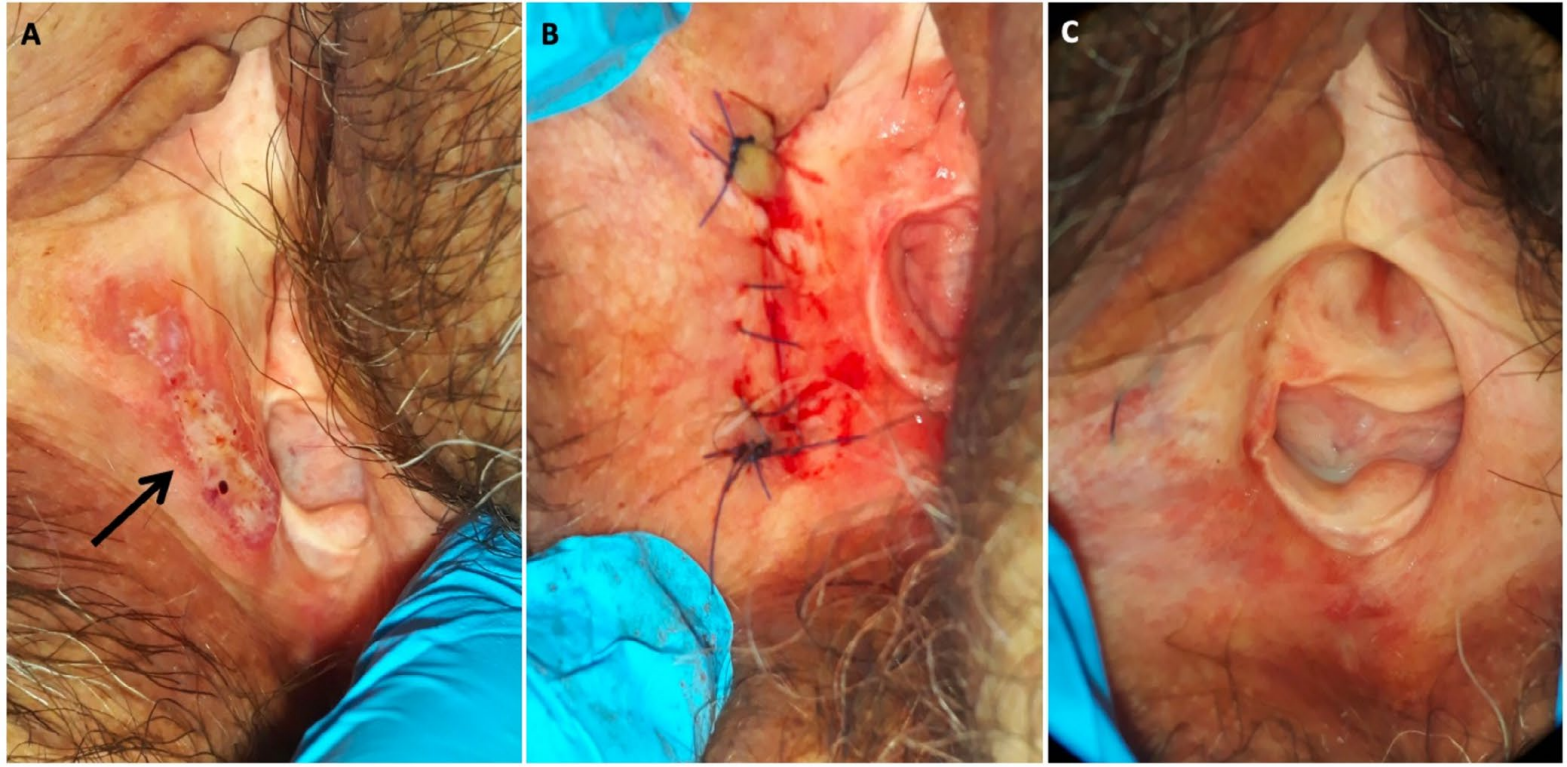
Clinical presentation. (A) Flat macular lesion in the lower third of the right hemivulva. (B) Local resection. (C) Thirty months of follow‐up.

A gynecological exploration revealed a flat, macular lesion measuring 3 × 4 cm that was identified in the lower third of the right hemivulva. The lesion was light brown with slightly darker central pigmentation and showed no rippled or wavy surface pattern. The borders were well defined and minimally raised. It was not ulcerated and exhibited well‐defined, slightly raised edges, with no bleeding or pain upon palpation (Figure 1A). A complete total body skin examination revealed no similar macular hyperpigmented lesions elsewhere. The vagina was free of visible lesions, while the cervix appeared atrophic and flattened. Colposcopy revealed no changes following Hinselmann's 5% test application.

## 3 Examination

**FIGURE 2:**
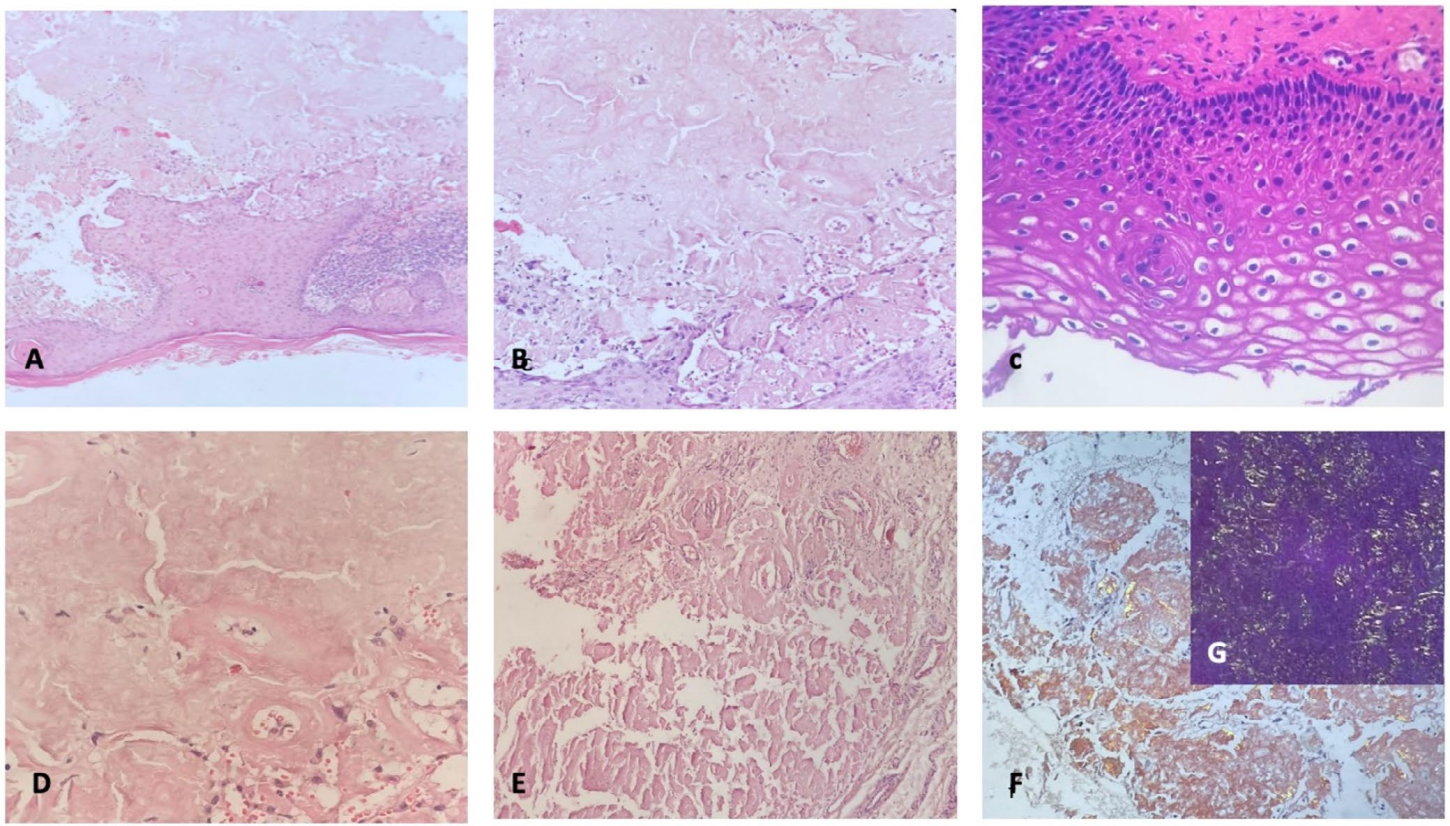
(A) Acanthosis, papillomatosis, and focal coilocytic changes suggestive of human papillomavirus (HPV) infection with classic appearance of subepithelial amyloid deposits (hematoxylin and eosin (H&E), ×4). (B) Higher magnification (H&E, ×40). (C) Koilocytosis (H&E, ×40). (D) Amyloid deposits in papillary dermis of vulvar skin (H&E, ×40). (E) Congo red‐staining (×10). (F, G) Apple‐green birefringence of amyloid deposits under polarized light (×10).

Pap smear samples were obtained from the cervix, and an incisional biopsy of the vulvar lesion was performed after infiltration of local anesthesia without complications. PAP smear test was negative for intraepithelial lesions or malignancy. The biopsy revealed amyloid deposits in the papillary dermis associated with HPV, after which immunohistochemical staining with Congo red was performed, showing apple‐green birefringence under polarized microscopy (Figure 2). HPV genotyping was also conducted from the paraffin‐embedded block with the Anyplex II HPV28 Detection kit (Seegene Inc), using a real‐time PCR that identifies 28 genotypes of low and high oncogenic risk, reporting positivity for genotype 40. Due to this diagnosis, evaluations were indicated to rule out systemic amyloidosis, which is the most common type. This evaluation included cardiologic evaluation with electrocardiogram and echocardiography to rule out cardiac involvement, nephrological study with renal function tests and urinalysis, and pulmonary and otolaryngologic evaluations to exclude amyloid deposits in the respiratory and upper airway mucosa, thereby confirming the diagnosis of localized disease.

## 4 Treatment and Follow‐Up

Given the patient's complaints of discomfort and pruritus, the lesion's unusual clinical appearance, and the absence of a standardized treatment for PLCA, the possibility of surgical resection was discussed in a tumor board after obtaining informed consent. A local vulvar resection was performed under sedation with clear margins (Figure 1B), which confirmed the diagnosis of primary vulvar cutaneous amyloidosis related to HPV, with follow‐up scheduled every 6 months. At 33 months post‐surgery, the patient remains in complete remission, showing no signs of recurrence or systemic involvement (Figure 1C). This case highlights the importance of considering localized cutaneous amyloidosis in the differential diagnosis of vulvar lesions, especially when associated with HPV.

## 5 Discussion

In this case, a 73‐year‐old woman presented with a solitary vulvar lesion that was histologically confirmed as a PLCA, which is associated with HPV. Immunohistochemically, Congo red staining demonstrated a classic apple‐green birefringence under polarized light, and systemic amyloidosis was ruled out by a multidisciplinary clinical evaluation. To our knowledge, this is one of the few documented cases of vulvar PLCA linked to a low‐risk HPV genotype without concurrent intraepithelial neoplasia. The rarity of this case lies in the detection of HPV genotype 40, a low‐risk type, in the affected tissue through molecular genotyping. PLCA of the vulva is a rare disease due to its clinical and histological heterogeneity, characterized by localized amyloid deposition in the dermis. Whether the presence of low‐risk HPV represents an incidental finding or a true trigger for localized amyloid deposition remains uncertain. Although a causal relationship cannot be definitively established, the absence of VIN and the presence of koilocytosis changes suggest that HPV‐related keratinocyte injury may serve as an epithelial trigger for amyloidogenesis, consistent with the keratin‐derived nature of amyloid observed in PLCA. The nodular subtype, which is the most diagnostically challenging, requires exclusion of systemic disease among the PLCA variants and is the least frequent and most difficult variant to diagnose [8]. Its presentation usually simulates neoplastic or chronic inflammatory conditions, emphasizing the need for histopathological confirmation and a thorough systemic evaluation [9].

Some authors suggest a possible link between HPV infection and cutaneous amyloid deposition in the genital tract, demonstrating amyloid deposits associated with vulvar intraepithelial neoplasia (VIN) of the usual type and positivity in 74% for high‐risk genotypes such as HPV 16 or 51. In addition, amyloid could be associated with regression or a better prognosis, since cases with amyloid had fewer relapses than those without [5, 7]. In contrast, our case was linked to a low‐risk genotype, with no association with neoplastic changes or high‐grade VIN, suggesting that HPV infection, without epithelial dysplasia, can trigger keratinocyte degeneration and subsequent amyloid deposition. This is consistent with the keratin‐derived epithelial origin of amyloid described in recent studies [5, 7].

Although excision is not routinely indicated for PLCA, in cases with symptomatic lesions and diagnostic uncertainty, surgical removal may serve both therapeutic and diagnostic purposes, particularly when malignancy cannot be ruled out clinically. Furthermore, the patient has remained disease‐free for 33 months after the complete excision of the lesion with clear margins. This result supports the hypothesis suggested by Islam et al. [7], who reported that amyloid in VIN could be related to a lower recurrence and a more indolent clinical course. Although our case does not present with concurrent VIN, the prolonged remission could indicate a relevant role of HPV‐induced epithelial stress and localized immune responses in amyloidogenesis. This could be an epithelial trigger without requiring dysplastic evolution.

Previous series have highlighted the link between vulvar amyloid deposition and usual type VIN, with amyloid present in up to 74% of cases and often associated with high‐risk HPV genotypes, particularly HPV 16 and 51. In these reports, amyloid deposition has been suggested as a marker of regression or a less aggressive clinical course. In contrast, this patient showed neither neoplastic changes nor high‐risk HPV infection, supporting the idea that low‐risk HPV alone may cause epithelial stress sufficient to produce keratin‐derived amyloid [6, 7]. Furthermore, only isolated case reports describe amyloidosis occurring in the vulva without VIN, and none have definitively confirmed low‐risk HPV at the molecular level in this context [5].

Given the rarity of vulvar PLCA and the limited reports associated with HPV, particularly those of low oncogenic risk, this case contributes to broadening the understanding of various cutaneous conditions of the lower genital tract linked to HPV and emphasizes a less recognized non‐dysplastic pathway potentially related to HPV‐induced epithelial injury. Additionally, it may enhance comprehension of vulvar HPV infection as a potential non‐dysplastic trigger for amyloidogenesis and reinforce the integration of PLCA in the differential diagnosis of vulvar lesions, highlighting the value of molecular diagnosis and the necessity of surgical excision and long‐term follow‐up as effective strategies.

## Author Contributions

**Jorge Hoegl:** conceptualization, data curation, formal analysis, investigation, methodology, project administration, writing – original draft. **Andreina Fernandes:** conceptualization, data curation, methodology, supervision, writing – review and editing. **Daniel Marquez:** supervision, validation, writing – review and editing. **Ysleyer Silva:** data curation, investigation, supervision, validation.

## Funding

The authors have nothing to report.

## Consent

Written informed consent was obtained from the patient for publication of this case report and any accompanying images. A copy of the written Consent is available for review by the Editor‐in‐Chief of the journal.

## Conflicts of Interest

The authors declare no conflicts of interest.

## Data Availability

Data available on request from the authors.
